# Crystal-Chemical and Thermal Properties of Decorative Cement Composites

**DOI:** 10.3390/ma14174793

**Published:** 2021-08-24

**Authors:** Vilma Petkova, Ventseslav Stoyanov, Bilyana Kostova, Vladislav Kostov-Kytin, Alexander Kalinkin, Irina Zvereva, Yana Tzvetanova

**Affiliations:** 1 Institute of Mineralogy and Crystallography “Academician Ivan Kostov”, Bulgarian Academy of Sciences, Academic Georgi Bonchev Street, Building 107, 1113 Sofia, Bulgaria; vkytin@clmc.bas.bg (V.K.-K.); yana.tzvet@gmail.com (Y.T.); 2 Faculty of Construction, University of Structural Engineering and Architecture “Lyuben Karavelov”, 175 Suhodolska Street, 1373 Sofia, Bulgaria; vensy.stoyanov@vsu.bg; 3 Academy of the Faculty of Fire Safety and Civil Protection, 171 Pirotska Street, 1309 Sofia, Bulgaria; 4 Department of Natural Sciences, New Bulgarian University, 21 Montevideo Street, 1618 Sofia, Bulgaria; bkostova@nbu.bg; 5 Kola Science Centre, Tananaev Institute of Chemistry and Technology of Rare Elements and Mineral Raw Materials, Russian Academy of Sciences, 14 Fersman Street, 184209 Apatity, Russia; a.kalinkin@ksc.ru; 6 Center for Thermal Analysis and Calorimetry, Staint-Petersburg State University Research Park, 26 Universitetskij Prospekt, 198504 Peterhof, Russia; irina.zvereva@spbu.ru

**Keywords:** white Portland cement, marble powder, cement replacement materials, thermal properties, reaction mechanism

## Abstract

The advanced tendencies in building materials development are related to the design of cement composites with a reduced amount of Portland cement, contributing to reduced CO_2_ emissions, sustainable development of used non-renewal raw materials, and decreased energy consumption. This work deals with water cured for 28 and 120 days cement composites: Sample A—reference (white Portland cement + sand + water); Sample B—white Portland cement + marble powder + water; and Sample C white Portland cement + marble powder + polycarboxylate-based water reducer + water. By powder X-ray diffraction and FTIR spectroscopy, the redistribution of CO_3_^2−^, SO_4_^2−^, SiO_4_^4−^, AlO_4_^5−^, and OH^−^ (as O-H bond in structural OH^−^ anions and O-H bond belonging to crystal bonded water molecules) from raw minerals to newly formed minerals have been studied, and the scheme of samples hydration has been defined. By thermal analysis, the ranges of the sample’s decomposition mechanisms were distinct: dehydration, dehydroxylation, decarbonation, and desulphuration. Using mass spectroscopic analysis of evolving gases during thermal analysis, the reaction mechanism of samples thermal decomposition has been determined. These results have both practical (architecture and construction) and fundamental (study of archaeological artifacts as ancient mortars) applications.

## 1. Introduction

Getting Portland cement is expensive, with high energy and raw material consumption production. A problem in its production is also the generation of carbon dioxide emissions [1,2]. In this regard, the advanced trends in building materials appeared to join the circular economy. This economic system aimed reduction of waste and carbon dioxide emissions and the continual use of resources, thus enhancing innovation and competitiveness [3,4].

The advanced tendencies in building materials development are related to the design of cement composites with a reduced amount of Portland cement, which will contribute to reducing CO_2_ emissions and sustainable development of non-renewal raw materials used and energy as well. This is achieved through cement replacement materials as natural pozzolans, fly ash, blast furnace slag, silica fume, metakaolin, carbonate rock (limestone, marble), etc. [2,5,6].

The interest in carbonate rocks as a cement substitute material is justified because their use reduces the total amount of clinker required to produce a certain amount of cement while increasing the total volume of cement obtained. That would result in a substantial amount of energy saving in the production of cement as the consumption of natural raw materials and the fuel needed for the clinker production would be reduced. Additionally, the reduction of CO_2_ emissions would achieve [2]. Such cement composite has applications for decorative stamps, balustrades, restoration of architectural monuments, etc. [7,8,9]. On the other hand, its use requires large amounts of another natural resource, namely water, compared to conventional cement. The increased amount of water implies the appearance of crystal-chemical changes in such composite cements [7,10]. The amount of water used, can be controlled by adding a polycarboxylate-based water reducer to the composite. That additive leads to steric repulsion of fine particles [10,11]. Due to the superior water-reduction capabilities of such composite materials, the workability is improved, however, that may lead to an increase in the number of fine particles [11,12,13]. As a result, the hardened self-compacting mortar is obtained, characterized by high early-strength, smooth surfaces, and dense structure [12,13,14].

This work deals with cement composites of white Portland cement, marble powder with and without polycarboxylate-based water reducer. For comparison, a composite of Portland cement and quartz sand as cement replacement materials were used. Earlier investigations [15,16] of such composites revealed some difficulties in the identification of samples’ solid phases, which made it difficult to determine the generalized scheme of samples’ hydration and the reaction mechanism of their thermal decomposition. The present study aims to clarify previously unsolved issues, showing the differences in the samples’ reaction mechanisms depending on the used cement replacement material.

To achieve the purpose, the more precise powder X-ray diffraction measurements and new ones of samples after heating up to 1100 °C, additional thermal analysis with mass spectroscopic registration of evolving gases, and new Fourier Transform Infrared spectroscopic investigations have been performed. All the methods used provided new results on the crystal-chemical and thermal properties of the samples (detailed scheme of their thermal decomposition mechanism).

Such knowledge is essential for obtaining good physical, chemical, and mechanical performance of composites with application in architecture and construction practice. The achieved results also are of fundamental importance in the study of archaeological materials—ancient mortars.

## 2. Raw Materials Composition and Samples Preparation

Used raw materials for sample preparation are white Portland cement (manufacturer, city), two types of aggregate (marble powder and sand), polycarboxylate-based high range water reducer (HRWR), and distilled water. The chemical and mineral composition of the white Portland cement and the used aggregates are presented in Table 1.

The white Portland cement—CEM I 52.5 N, produced by Devnya Cement (Devnya, Bulgaria). The mineral composition is calculated by Bogue method [17].

The first used aggregate is marble powder, produced by the company White Marble Products AIAS S.A. (Nikisiani, Greece).

The second used aggregate is sand—clean washed and dried river sand with over 85.0% content of SiO_2_—quartz. The aggregate is presented by fineness spheroid modulus—2.7 (EN 12620:2002 + A1:2008/HA:2008) with shape index—4.6% (EN 933-4:2008).

The used HRWR (Sika ViscoCrete 5-800, Sofia, Bulgaria) is in dosage 2.0% by weight of white cement. This HRWR is chloride-free, soluble in water, without any retarding effects, and a density of 1.07 g/cm^3^ (at 20 °C).

The three cement composite samples have been prepared. Sample A, a reference (white Portland cement + sand + water), with composition satisfying the EN 196-1 (Section 6) requirements [18] for the determination of the cement compressive strength. Sample B (white Portland cement + marble powder + water) was prepared by mixing procedure, according to the EN 196-1 [18], with prolonged time of homogenization at high speed (to obtain self-leveling properties or 120 s). The composition of sample B was determined by optimization to achieve high-quality smooth surfaces of the hardened samples [19]. This procedure was applied to the data obtained from experimental tests (D-optimal plan with constant cement-to-aggregate ratio). The sample C (white Portland cement + marble powder + HRWR + water) was prepared according to the EN 196-1, with the same cement-to-aggregate ratio as sample B and with the maximum possible amount of water, where the sedimentation or segregation borders were not observed 10 min after mixing.

The three samples were molded without any compacting treatment in 6 prisms (two prisms for each sample), 40 mm × 40 mm × 160 mm, for 1 day in a moist atmosphere (>95% RH and 20 °C). Then the unmolded samples were stored underwater (20 °C) until strength testing (28 and 120 days) [20,21]. The samples A, B, and C undergoing water curing for 28 days are named A028, B028, C028, and those for 120 days—A120, B120, and C120. The components ratio in studied samples is presented in Table 2.

The same samples have been investigated earlier by us where their physical-mechanical properties—bulk density after immersion, adsorption after immersion, compressive strength, and pore volume were defined well. The composites with marble powder show denser structures with the tendency of self-desiccation. The composite low pore volume and denser structure are demonstrated by the Scanning electron microscopy results, as well. The use of polycarboxylate-based additive decreases the water amount and improves the fine particle dispersion and hardness respectively [15,16].

## 3. Experimental Methods

The time-resolved high-temperature powder X-ray diffraction (HT-PXRD) measurements were collected at PANalytical Empyrean (Malvern Panalytical, Malvern, UK) equipped with a multichannel detector (Pixel 3D) (Malvern Panalytical, Malvern, UK) using (Cu Kα radiation 45 kV–40 mA) radiation in the 10–80° 2θ range, with a scan step of 0.026° for 33 s. Phase identification has been performed using various search-match programs software as well as data from the Inorganic Crystal Structure Database (ICSD) [22].

FTIR measurements are performed by FTIR Spectrometer, (Varian 660, Agilent Technologies, Wien, Austria), covering the range of 400–4000 cm^−1^. The samples were prepared as pellets consisting of low-dispersed KBr and powder of prepared mixture. The transmittance spectra were collected using an MCT detector with 64 scans and 1 cm^–1^ resolution.

TG/DTG-DSC analysis was carried out on a Setsys Evolution 2400, (SETARAM, Lion, France), combined with an OmniStar mass-spectrometer (Pfeiffer Vacuum GmbH, Wetzlar, Germany), in the temperature range room temperature (RT)—1100 °C; in gas medium—static dry air atmosphere, with a heating rate of 10 °C/min. The operational conditions of the TG/DTG–DSC-measurements were: sample mass of 15.0 ± 1.0 mg (mass resolution of 0.02 µg) and alumina sample crucible with a volume of 100 µL.

## 4. Results and Discussion

### 4.1. PXRD Phase Analysis

**Figure 1:**
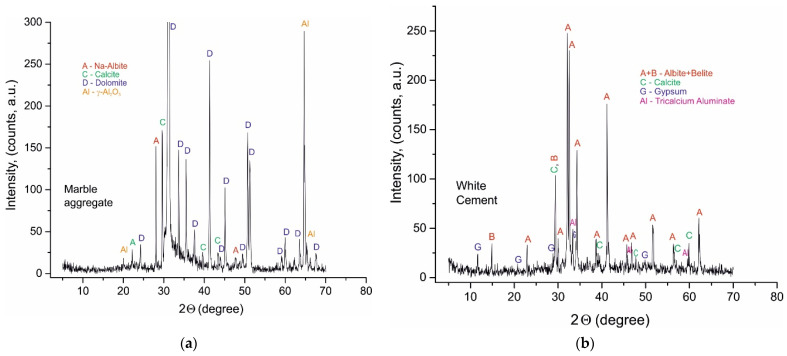
(**a**) PXRD pattern of marble powder aggregate. (**b**) PXRD pattern of white Portland cement.

**Figure 2:**
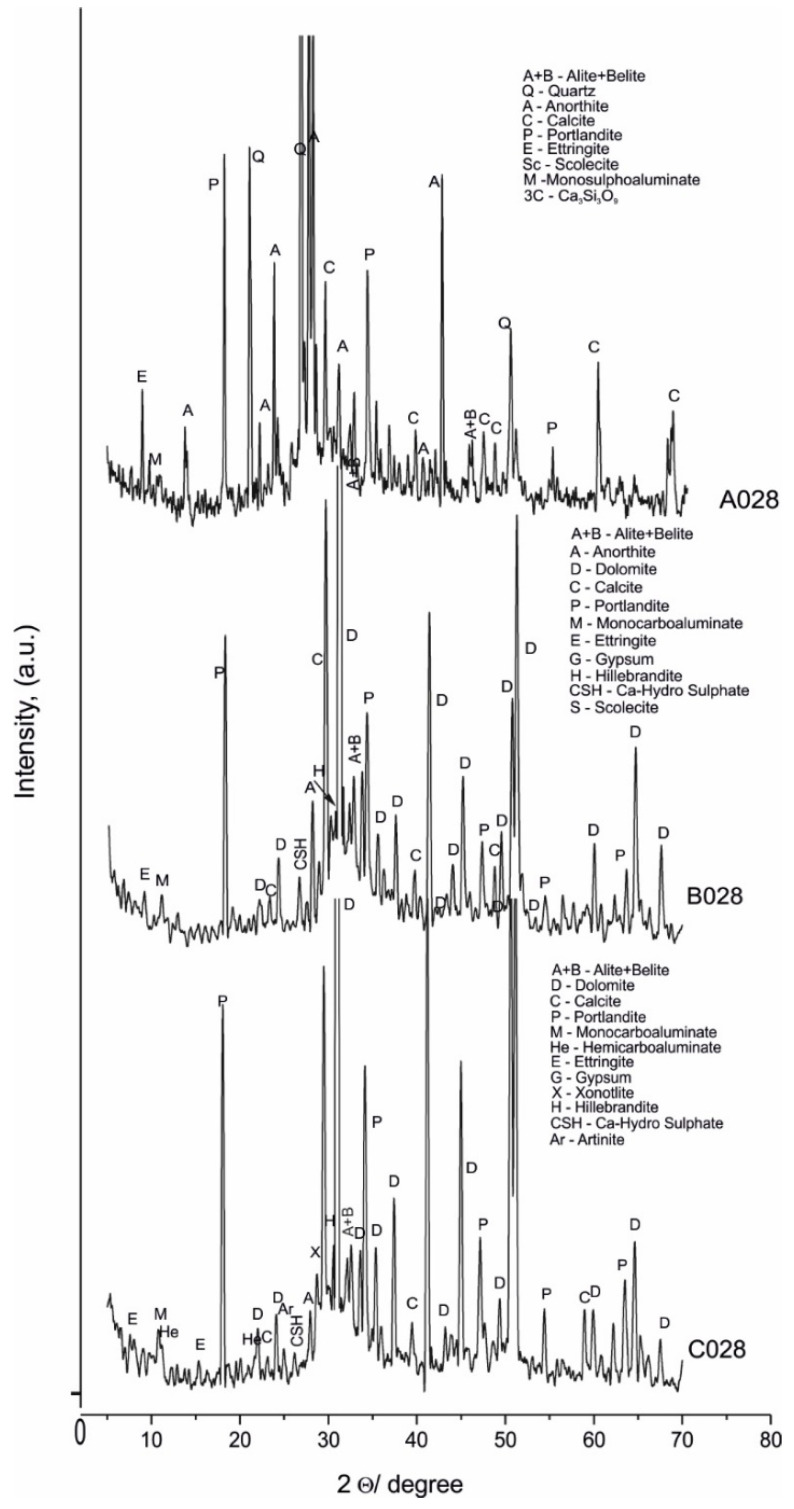
PXRD patterns of A028, B028, and C028.

**Figure 3:**
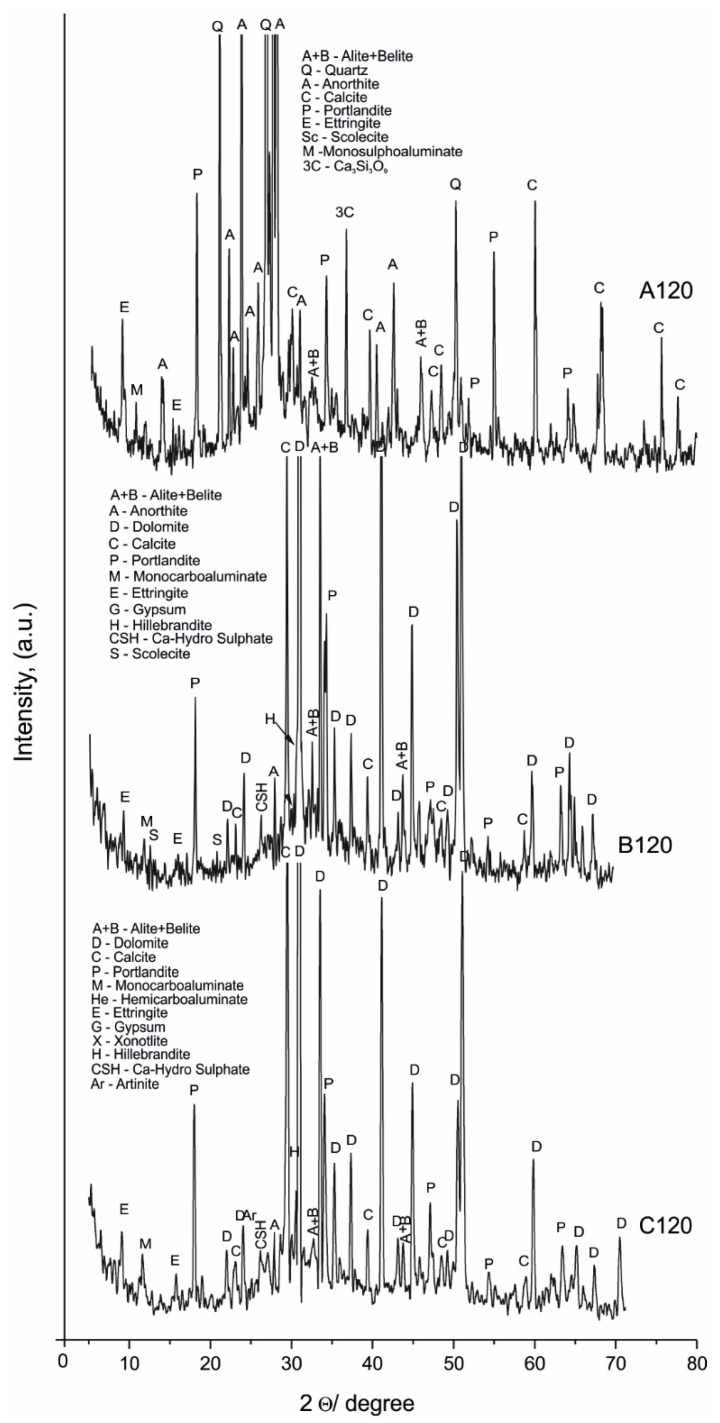
PXRD patterns of A120, B120, and C120.

The PXRD was used to better evaluate the investigated samples. The obtained results for raw materials and samples A028, A120, B028, B120, C028, and C120 are shown in Figure 1, Figure 2 and Figure 3 and Table 3.

The defined mineral phases in raw materials are belite, alite, tricalcium aluminate, gypsum, and calcite in white Portland cement; dolomite and Mg-rich calcite in marble powder aggregate; quartz in sand aggregate.

The phase composition of water cured samples is determined by the presence of the raw cement minerals, marble powder aggregate, HRWR additive, water-to-cement ratio, and overall ratio of the main components in the samples [23].

The PXRD analysis (Table 3, Figure 1, Figure 2 and Figure 3) reveals the presence of two groups of minerals in the studied samples: (i) relict minerals from the raw materials: belite, alite, calcite, quartz, dolomite; and (ii) newly formed minerals: portlandite, hemi- and mono-carboaluminate, ettringite, monosulphoaluminate, hillebrandite, scolecite, xonotlite, calcium hydrogensulphate, and artinite.

The obtained results justify the distribution of the new phases into several groups as follows:

- containing OH^−^ and HCO_3_^−^/CO_3_^2−^;
- containing OH^−^ and HSO_4_^−^/SO_4_^2−^;
- containing OH^−^ in hydrate phase and OH^−^ in hydrosilicates formed of main oxides CaO, Al_2_O_3_, SiO_2_.

Their distribution is presented in detail in Table 3.

The identification of mono- (B120 and C120) and hemi-carboaluminate (B028 and C028) [24] as well as spurrite [25,26,27,28] (B120 and C120) evidence the contribution of carbonate ions introduced in the system from the marble powder aggregate. The identification of ettringite and monosulphoaluminate [17,29,30] evidence the contribution of sulphate ions from the raw white Portland cement after hydration.The PXRD patterns are dominated by the reflexes of dolomite, calcite, belite, alite. The newly formed post-hydration phases are characterized by lower peak intensity, predetermined by the lower crystallites size, especially pronounced for samples with 28 days of curing. Probably, the lower water-to-cement ratio mostly influences the formation of hydrate phases with small crystallites. This is confirmed by the detected broadening of their reflections (Figure 2). Thus, microscopic, spectroscopic, and thermal analyzes have also been performed to confirm and refine these results.

The obtained new PXRD results do not confirm the previous assumption of the isomorphic substitution for carbonate ions in ettringite [15]. They show the redistribution of carbonate ions, as well and sulphate ions in new solids, proven by the detection of spurrite, monosulphoaluminate, calcium hydrogensulphate, and artinite.

Table 4 shows the results of the PXRD analysis of samples solid residue after heating up to 1100 °C.

The identified phases prove the conversion of the hydrated into anhydrous calcium-aluminum silicates after thermal heating to 1100°C. Such results are related to the realized complete dehydration, dehydroxylation, as well as incomplete decarbonation and desulphuration of the hydrate, carbonate-containing, and sulfate-containing phases (Table 4). The sample A solid residue composition includes: (i) high-temperature silicate phases with SiO_3_-structure (anorthite and wollastonite), and SiO_4_-tetrahedra (larnite); (ii) ternesite and anhydrite, incorporating part of the sulphate sulphur. The solid residue of samples B and C contains anhydrous silicates (wollastonite, larnite, anorthite, and akermanite) and calcium aluminum oxide, result from high-temperature solid-phase synthesis at oxidizing atmosphere. Ternesite and/or anhydrite and/or spurrite were also registered.

### 4.2. FTIR Spectroscopy

**Figure 4:**
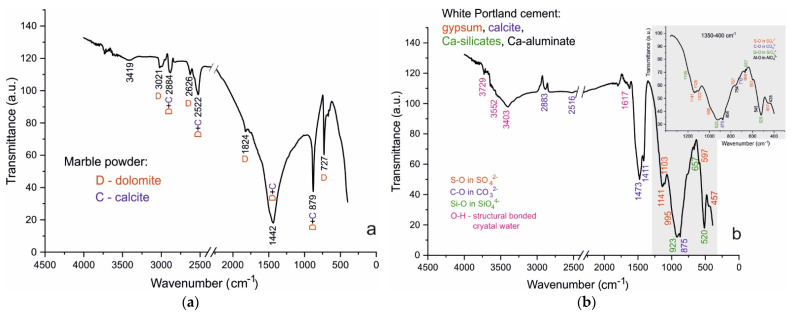
(**a**) FTIR spectra of raw materials marble powder; (**b**) FTIR spectra of raw materials white Portland cement. Insertion: magnification of spectral range 1350–400 cm^−1^ for white Portland cement.

**Figure 5:**
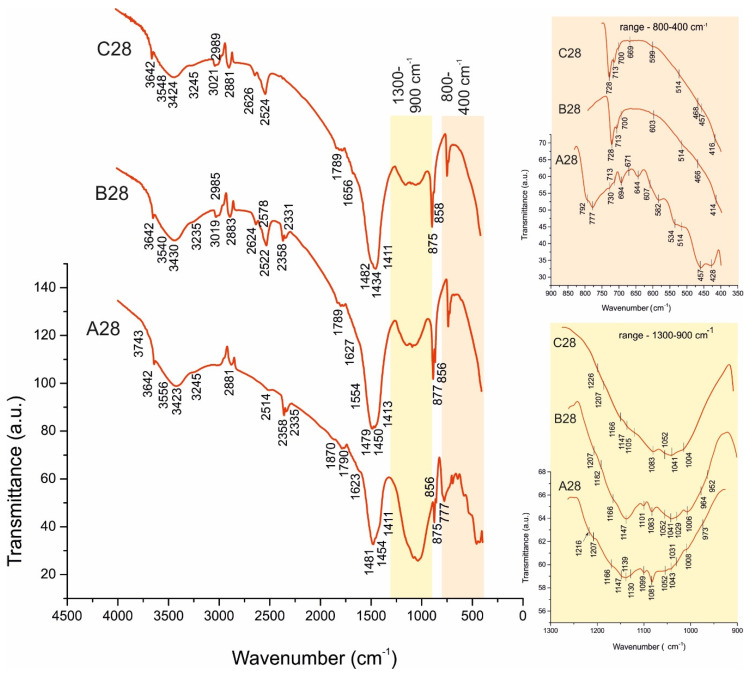
FTIR spectra of A028, B028, and C028. Magnifications: spectral ranges 1300–900 cm^−1^, and 900–350 cm^−1^.

**Figure 6:**
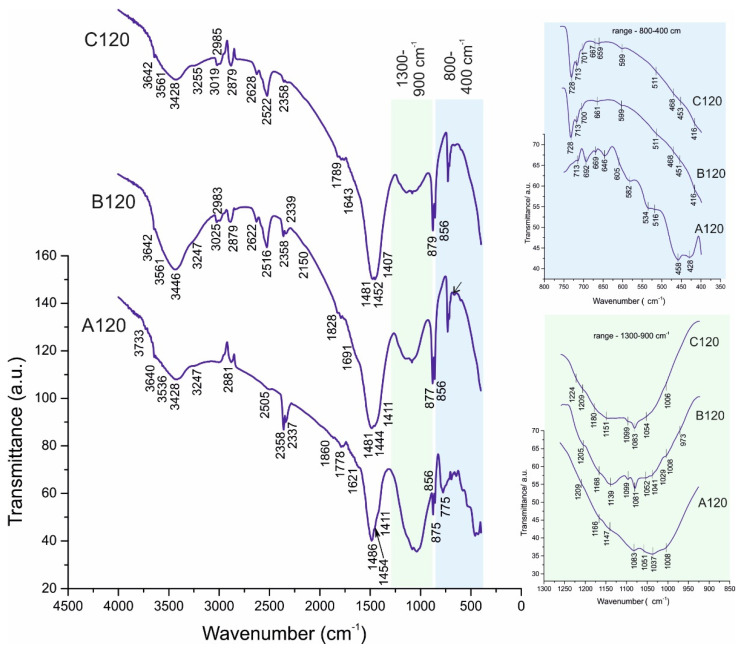
FTIR spectra of A120, B120, and C120. Magnifications: spectral ranges 1300–850 cm^−1^, and 800–350 cm^−1^.

The results obtained by FTIR spectroscopy (Table 5) of the raw marble powder and white Portland cement (Figure 4), as well as of the studied samples (Figure 5 and Figure 6) complement the results of the PXRD analysis. The FTIR spectroscopy is a suitable method for the investigation of cement composites allowing identify the solids in minimum quantities and fine dispersity [27].

O-H structural ^(1)^—O-H bond in structural OH^-^ anion [28,29]

O-H crystal ^(2)^—O-H bond belonging to crystal bonded water molecules [28,29] 

According to the results of PXRD analysis and FTIR spectroscopy, the following reaction mechanism scheme of samples hydration have been defined: 

#### 4.2.1. Sample A

2Ca_3_SiO_5_ (C_3_S) +**7**H_2_O → Ca_3_Si_2_O_7_.4H_2_O (C-S-H gel) + 3Ca(OH)_2_ (fast) (1)

2Ca_2_SiO_4_ (C_2_S) + 5H_2_O → Ca_3_Si_2_O_7_.4H_2_O (C-S-H gel) + Ca(OH)_2_ (slow) (2)

Ca_3_Al_2_O_6_ (C_3_A) + 3CaSO_4_.2H_2_O + 26H_2_O → Ca_6_Al_2_(SO_4_)_3_(OH)_12_.26H_2_O (Ettringite) (3)

Ca_6_Al_2_(SO_4_)_3_(OH)_12_.26H_2_O + 2Ca_3_Al_2_O_6_ (C_3_A) + 4H_2_O→3Ca_4_Al_2_(OH)_12_SO_4_.6H_2_O (4)

Ca_3_Al_2_O_6_ (C_3_A) + 3CaSO_4_.2H_2_O + 8H_2_O→Ca_4_Al_2_(OH)_12_SO_4_.6H_2_O + Ca(HSO_4_)_2_ + Ca(OH)_2_ (5)

CaCO_3_ + CO_2_ + H_2_O → Ca(HCO_3_)_2_ (6)

Ca_3_SiO_5_ (C_3_S) + 2H_2_O → Ca_2_SiO_3_(OH)_2_ + Ca(OH)_2_ (7)

Ca_3_Al_2_O_6_ (C_3_A) + 3SiO_2_ + 5H_2_O → CaAl_2_Si_3_O_10_.3H_2_O + 2Ca(OH)_2_ (8)

Equations (1)–(3) are essential for the reaction mechanism of cement clinker hydration with the formation of hydrated phases (C-S-H gel) [17] for sample A. The ettringite formation takes place in the early stages of cement minerals hydration (Equation (3)). With a residue of C_3_A, ettringite participates in the crystallization of mono-sulphate aluminate (Equation (4)). It is assumed, together with the formation of mono-sulphate aluminate (Equation (4)), is a possible parallel reaction in which a certain amount of Ca(HSO_4_)_2_ is formed (Equation (5)). It is possible, under hydration conditions, the relict calcite partially transforms to Ca(HCO_3_)_2_ (Equation (6)). It is also possible crystallization of scolecite and hillebrandite, (Equations (7) and (8)) when Ca, Al, and Si-ions content, with origin raw materials (cement clinker and quartz sand as aggregate—sample A). During most reactions, portlandite was also formed. The presentation of the hydration reaction mechanism was by a complex of Equations (1)–(8) [1,11,18].

#### 4.2.2. Samples B and C:

The essential 1, 2, 3, and Equations (6)–(8) describe the hydration mechanism of samples B and C. For the difference from sample A, for samples B and C, the Equation (9) was added and Equations (4) and (5) were replaced by Equations (10) and (11) (due to different aggregate—marble powder). The use of marble powder (dolomite) increases the carbonate ions content in the cement mortars [26,39,40,41], which is a prerequisite for the formation of carbonate-containing minerals such as hemi-, mono-carboaluminate, and Ca(HCO_3_)_2_ by the Equations (6), (10) and (11):

2Ca_3_SiO_5_ (C_3_S) + 4SiO_2_ + H_2_O → Ca_6_Si_6_O_17_(OH)_2_ (9)

Ca_3_Al_2_O_6_ (C_3_A) + 0.5CaCO_3_ + 0.5CaO + 12H_2_O → Ca_4_Al_2_(OH)_13_(CO_3_)_0.5_.5.5H_2_O (10)

Ca_3_Al_2_O_6_ (C_3_A) + CaCO_3_ + 11H_2_O → Ca_4_Al_2_(OH)_12_(CO_3_).5H_2_O (11)

Additional evidence for the crystal-chemical properties of the investigated samples is presented by the results of thermal analysis with mass spectrometry analysis of evolving gases as a direct proof of the ongoing thermal reactions.

### 4.3. Thermal Analysis

**Figure 7:**
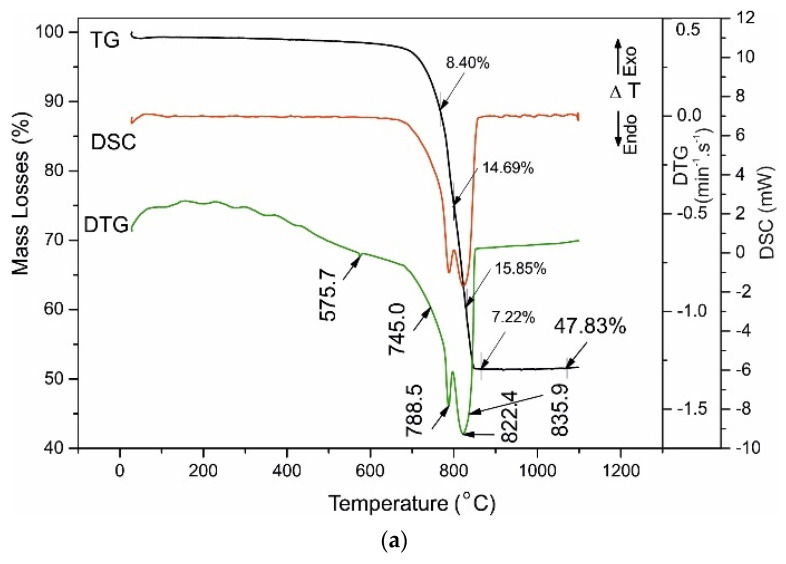

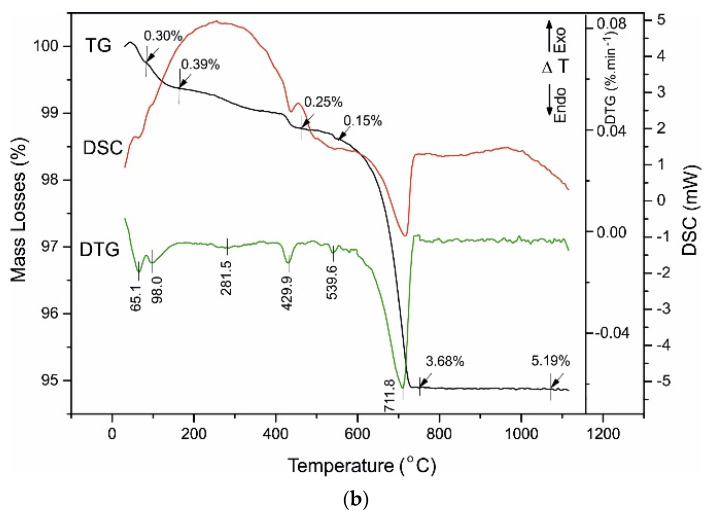
(**a**) TG-DTG-DSC curves of raw aggregate—marble powder. (**b**) TG-DTG-DSC curves of raw white Portland cement.

**Figure 8:**
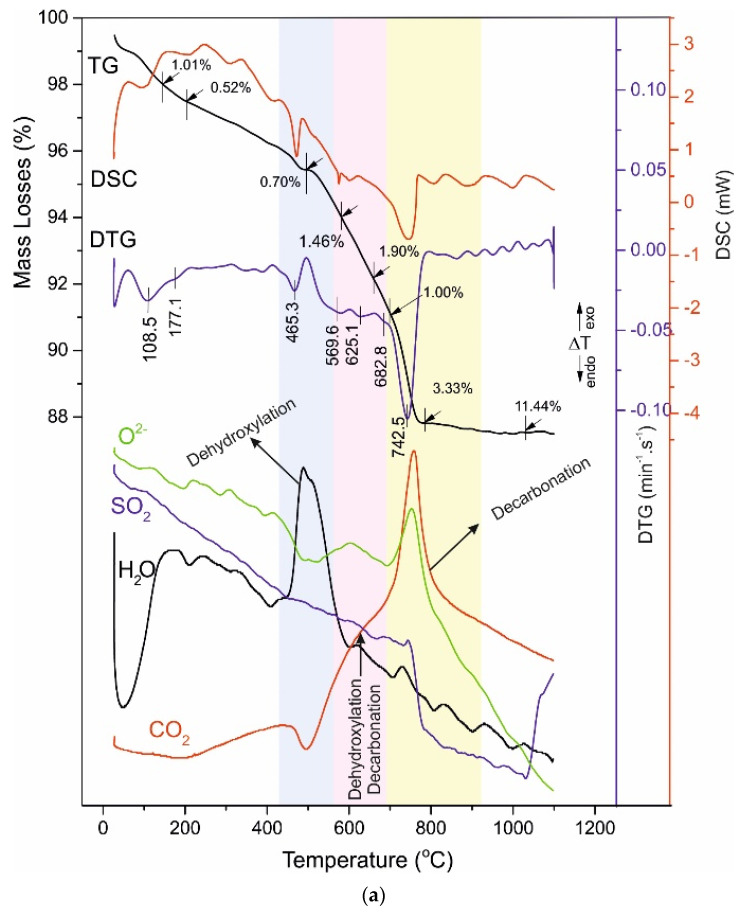

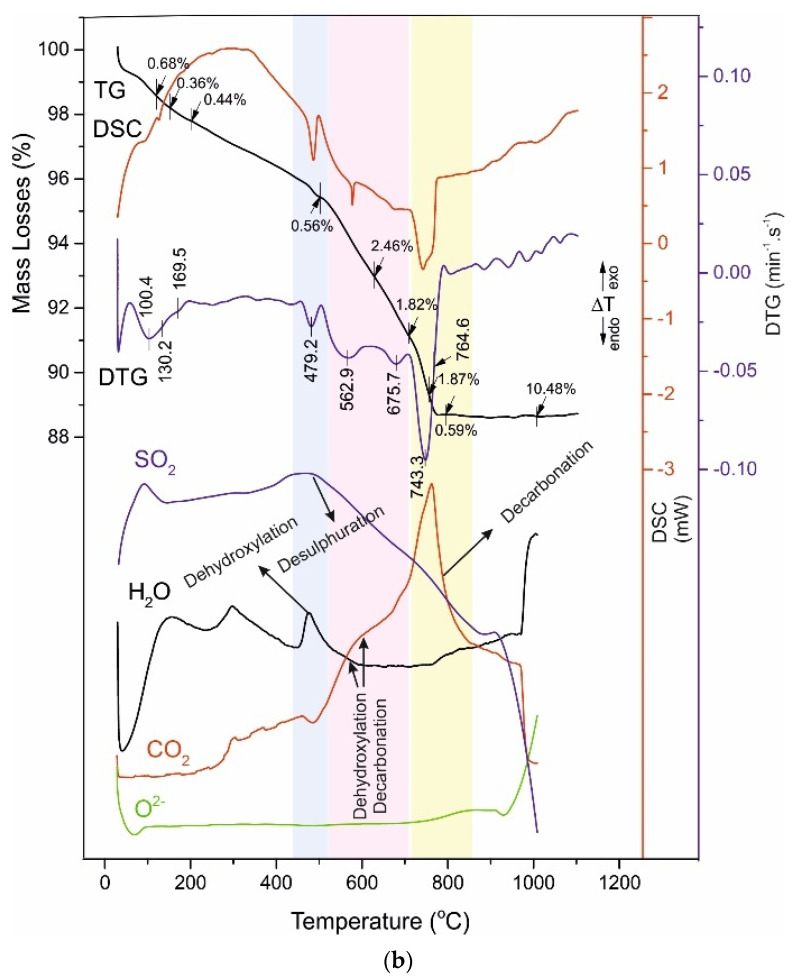
(**a**) TG-DTG-DSC curves of A028. (**b**) TG-DTG-DSC curves of A120. The temperature intervals in which thermal processes take place are marked with a different color background.

**Figure 9:**
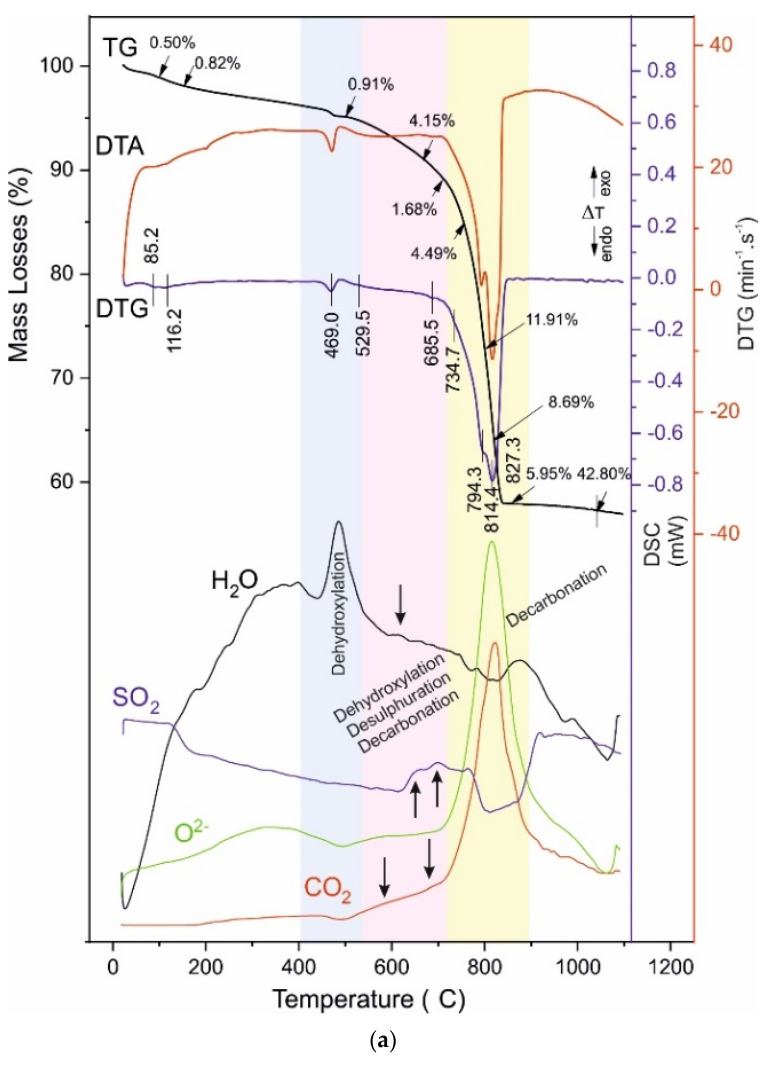

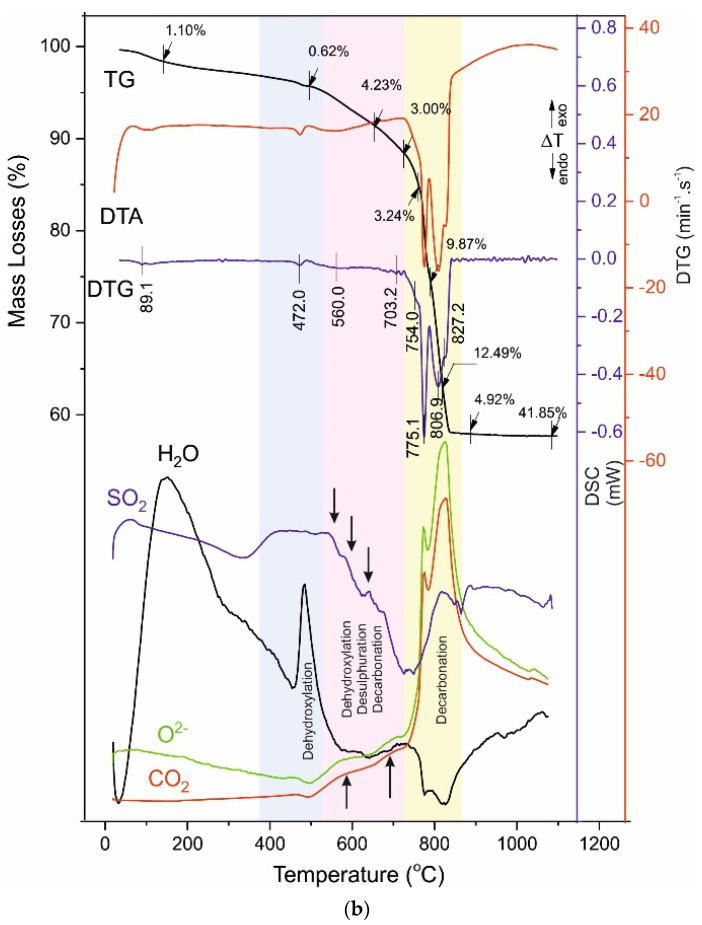
(**a**) TG-DTG-DSC curves of B028. (**b**) TG-DTG-DSC curves of B120. The temperature intervals in which thermal processes take place are marked with a different color background.

**Figure 10:**
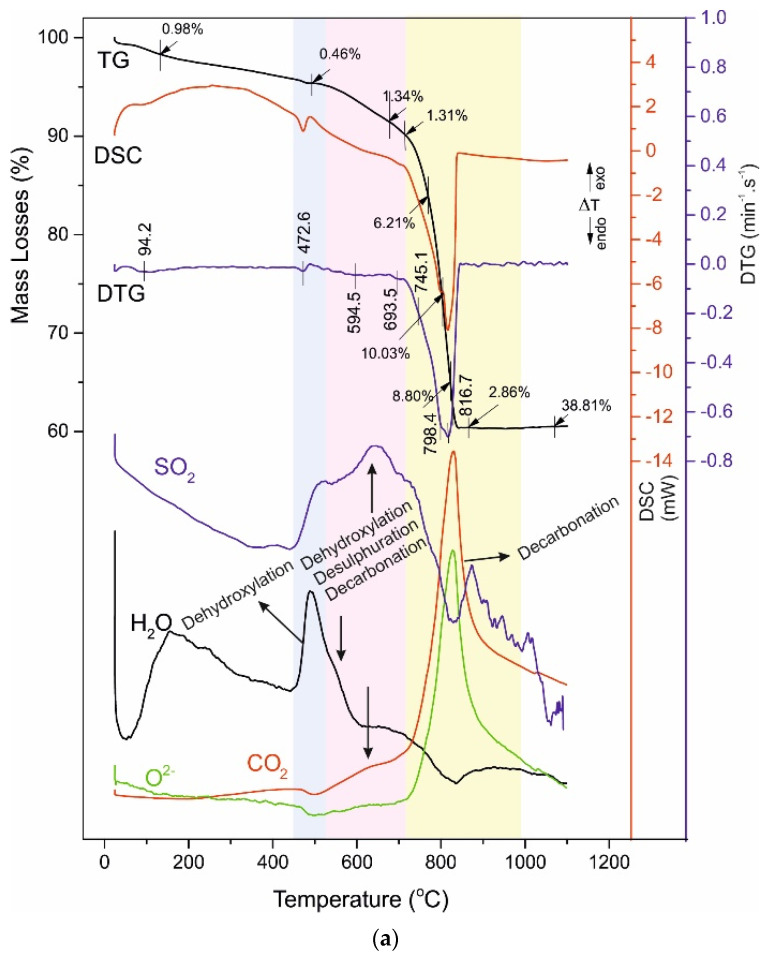

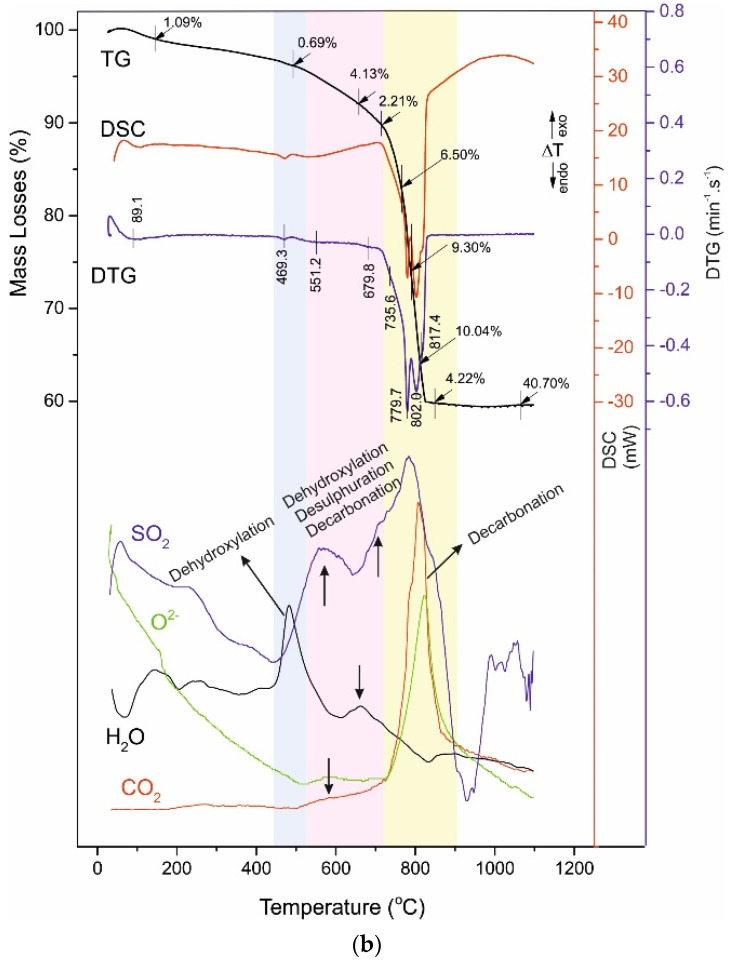
(**a**) TG-DTG-DSC curves of C028. (**b**) TG-DTG-DSC curves of C120. The temperature intervals in which thermal processes take place are marked with a different color background.

The obtained results are shown in Table 6 and Figure 7, Figure 8, Figure 9 and Figure 10, where Figure 7a is for the raw aggregate marble powder, Figure 7b—for raw white Portland cement, both presented for comparison with investigated samples: A028 and A120 (Figure 8), B028 and B120 (Figure 9), C028 and C120 (Figure 10).

Figure 7b shows the thermal behavior of Portland cement, characterized by total ML of just over 5% due to: (i) dehydration of crystal bonded water molecules (up to 220 °C); (ii) Ca(OH)_2_ and C-S-H gel dehydroxylation of structural OH- anions; and (iii) dolomite decabonation (most of the mass losses (ML)). 

Thermal decomposition of dolomite is a two-stage process and was carried out at temperatures of 740–860 °C with ML close to the theoretical ΔG^teor^ = 47.83%. Decomposition results in CaO and MgO in the solid decomposition residue and ML bind to CO_2_, and oxygen emissions in the gas phase [20].

Thermal analysis of marble powder shows the decarbonation performing into two well-defined stages (DTA peaks with T_infc_ at 806.6 °C and 821.2 °C), and two inserted steps with shoulders at 745.0 °C and 835.9° C. The total ML are about 45% which defines the dolomite and calcite as main carbonate minerals (Figure 7a, Table 3 and Table 6).

The thermal decomposition of studies samples is separated into four temperature ranges describing their thermal behaviour. The ML in these ranges are as follow (Table 5): I^st^ range—1.00–1.53%, II^nd^ range—0.46–0.91%, III^rd^ range—2.65–7.23%, and IV^th^ range—2.46–31.04%. The distribution of ML in the ranges is related to:

- I^st^ range, Dehydratation (40–250 °C) [13,19,42,43,44,45,46,47,48];
- II^nd^ range, Dehydroxylation (420–470 °C) [19,34,49,50];
- III^rd^ range, Dehydroxylation, uncomplete Decarbonation and uncomplete Desulphuration (520–730 °C) [19,34,49,50]
- IV^th^ range, uncomplete Decarbonation and Desulphuration (730–850 °C) [19,51,52,53,54,55,56,57,58,59,60,61].

*I^st^ range—Dehydratation (40–250 °C)*

At this range, the processes of dehydration of crystal bonded water molecules from the newly formed hydrate phases (samples A, B, and C) are carried out (Table 3 and Table 6). The registered low values of ML correspond to the small amount of crystal bonded water molecules. These results coincide with the low water-to-cement ratio of samples B and C (Table 2).

*II^nd^ range—Dehydroxylation (420–470 °C)*

The thermal effects measured in this narrow range are weak but well manifested and persistent in all samples. The characteristic of this temperature range is the realization of a distinct thermal reaction, which is of low intensity and with low ML (around and below 1%) (Table 6). 

Based on our earlier results [19], and data by the others [51,52,53,54,55,56,57,58,59], it is evidenced that the ML in this temperature range are associated with the portlandite dehydroxylation. The measured values of ML are lower in comparison to literature data, showing the lower quantity of portlandite crystals leading to the increased quantity of C-S-H phases. As a result, the samples obtain higher strength properties forming hydrated phases [19].

The ML decrease at samples A and B as follow: from 0.68% (A028) to 0.56% (A120), a decrease of 0.12%, and from 0.91% (B028) to 0.62% (B120)—decrease of 0.29%. The sample C shows the reverse trend—from 0.46% (C028) to 0.69% (C120)—increase of 0.23% (Table 6). These results show the enlarged expansion of C-S-H gel (with structurally bonded water molecules) at B120 due to the sample hydration, which coincides with the measured lowest values of pore volume and highest values of compressive strength [15]. The increase of ML at samples C with prolonged water curing does not correspond to the measured changes in pore volume and compressive strength [15]. Such behavior is due to the presence of HRWR additive, reducing the water-to-cement ratio (Table 2) without any changes in the values of compressive strength regardless of the presence of marble powder in the sample.

*III^rd^ range—Dehydroxylation,* uncomplete *Decarbonation, and* uncomplete *Desulphuration (520–730 °C)*

In this temperature range, the dehydroxylation of crystal bonded water molecules in hydrated phases (ettringite, monocarboaluminate, hillebrandite, etc.) occurs [48,50,51,52,53,54,55,56,57,58]. 

The measured mass losses of samples A and B show maintenance of the values as follow: a total of 4.36% for A028 and 4.28% for A120 (Table 6, Figure 8), as well as of 5.83% (B028) and 7.23% (B120) (Figure 9, Table 6). There are significant changes in the mass loss values for sample C—an increase from 2.65% (C028) to 6.34% (C120) (Figure 10, Table 6), which evidence changes in the phase composition and the distribution of crystal bonded water molecules. An increase of mass losses in this temperature range was found for the B series samples. That could be explained by the incorporation of larger amounts of hydroxyl, carbonate, and/or sulphate ions in the structure of B028 and B120. 

The mass losses via dehydration of portlandite and C-S-H phases in sample A at the II^nd^ and III^rd^ ranges are the smallest, and the ratio between them is close to 1:1 (mass losses A028:mass losses A120) (Table 6). 28 days of hydration of samples B and C decrease the mass losses in III^rd^ range from 5.83% (B028) to 2.65% (C028) (Table 6). That may be explained by the lower pore volume of B028 [15], which is necessary for the new C-S-H phases growth. The low content of the C-S-H phase in sample C is due to the very low water-to-cement and water-to-fines ratio (Table 2). 

The present studies evidence the decomposition processes in this temperature range are more complex. Mass-spectrometric analysis of the evolving gases revealed that except dehydroxylation of C-S-H phases, the decarbonation and desulphuration occur (Figure 8, Figure 9 and Figure 10). 

The thermal decomposition of A028, and A120, assumed that carbonate ions evolved as CO_2_ and O^2−^, sulphate ions as SO_3_ and O^2−^ (better expressed at A120), and the water vapor, identified as OH^−^ (Figure 8). The OH-ions registration evidences the simultaneous formation of the ettringite, scolecite, xonotlite, and portlandite during the hydration process. The evolving of CO_3_ and SO_4−_ions prove diversity in their redistribution in the solid phase. 

At the first three temperature ranges, an evaluation of the effect of the composition on the sample’s microstructure and their subsequent use in construction practice has been sought. This evaluation includes: determining changes in the amount of crystallization and structurally bound water, uncomplete decarbonation of carbonate and hydrocarbonate ions, and uncomplete desulphuration of sulphate ions in lower temperature regions, such as 520–750 °C. Registering of a decarbonation and desulphuration process at a lower temperature range, than that one known for traditional carbonate (730–850 °C) and sulphate minerals (1200–1500 °C) is considered. That probable evidence: (i) isomorphic substitution and structural changes, including carbonate and sulphate ions in the structure of minerals through weaker chemical bonds or (ii) or for intermediate thermal reactions with decomposition of hydrogen carbonate and/or hydrogen sulphate phases. The results of PXRD and FTIR do not confirm the formation of isomorphic phases, only prove the formation of hydrogen-containing phases. The hydrogen-containing phases decompose at lower temperatures, realizing sulphur oxides at lower than traditionally known temperatures.

In sample B, are registered CO_2_ and OH^-^, and SO_2_ (Figure 9). Sulphate- and carbonate-bearing minerals are stable at this temperature stage, which suggests that OH^-^, SO_x_, CO_2_, and O^2−^ detection is of their realization from phases in which they are included as defects, situated in different crystallographic positions. These defects can be associated with weaker inter-/intra bonds at interlayer spaces and channel structures [62,63,64], leading to structural compaction and physical-mechanical properties improvement [19,65,66]. The CO_2_ and OH^-^, and SO_2_ evolving are most distinct in B028 and B120 but are also observed at C028 and C120 (Figure 10) being less pronounced, because of the presence of HRWR additive reducing the water-to-cement ratio.

The dehydration-decarbonation reactions could also be attached to the III^rd^ range. They occur with the carbonate ions realization from the hydrogen-carbonate phases (CaHCO_3_, mono/hemi-carboaluminates) which are meta-stable and decarbonize at lower temperatures than CaCO_3_. That explains the low-intensity peaks insertion into the major decarbonation peak of samples in the next temperature range of 730–850 °C. The presence of HCO_3_^−^/HSO_4_^−^ explains the registration of hydroxyl and carbonate/sulphate ions in the evolving gas [67,68]. The HCO_3_^−^/CO_3_^2−^ equilibrium depends on the raw materials and environment: humidity, temperature, sulphur, nitrogen, carbon-containing, and other gases, pH, etc. The change in the ratio of the phases containing HCO_3_^−^/CO_3_^2−^ and HSO_4_^−^/SO_4_^2−^ ions is carried out at the solid-gas (material-to-atmosphere) interphase boundary. These processes lead to changes in the chemical activity and solubility of the materials and determine their durability, which is important for construction practice. 

*IV* *^th^ Decarbonation and Desulphuration (73* *0–85* *0 °C)*

The decomposition of carbonate-containing phases (Figure 8, Figure 9 and Figure 10, Table 6) of samples A, B, and C is performed at higher temperatures, compared to the raw Portland white cement and marble powder. Significant differences between the mass losses are measured (3.68% in the raw cement, 46.16% in the raw marble powder, and 30.06% in sample C120), defined by the different amounts of carbonate minerals. The temperature decomposition intervals are maintained without substantial changes. The marble powder decarbonation occurs with several parallel reactions at close temperatures, which results in the splitting of peaks in the DTG/DSC curves, seen at the samples B and C, as well.

The multi-step decarbonation process indicates the thermal decomposition of various carbonate-containing phases—dolomite, Mg-rich-calcite, and hydrated calcium-aluminates. Their decomposition causes the splitting of the peaks in the DTG/DSC dependencies and changes in their intensity. The curve describing the CO_2_ from the carbonate ions in the evolving gas analysis follows the course of the DTG/DSC peaks which is direct evidence of the overlapping thermal reactions in this interval.

The use of dolomitic marble powder as aggregate slightly increases the temperature of decarbonation with the benefit of the samples B and C (Table 6, Figure 9 and Figure 10). The most accurate explanation of this fact is the formation of dense structures, which affect the results of physic-mechanical analyses [15].

As a result of the thermal reactions in the IVth range, it is possible to obtain CaCO_3_ and CaSO_4_, as well as anhydrous Ca-, Ca-Mg-, and Ca-Al silicates. These are solid-phase thermal reactions that take place without ML. The PXRD analysis of the solid residual proves the formation of such phases (Table 4). In this temperature range, it is possible to form a new phase—spurrite, due to anhydrous calcium silicates, oxides, and CO_2_ (vapor), existing in the system [69,70]. The spurite synthesis was carried out during the decarbonation of dolomite, calcite, and Mg-rich calcite, when CO_2_ and O_2_ emitted as the gas phase, and Ca and Mg-oxides—fixed as solid phase [71,72,73,74]. Another possibility is the spurrite synthesis by a solid-phase reaction of oxides (CaO, SiO_2_), and/or silicates (CaSiO_3_, Ca_2_SiO_4_), and calcite. There exist data for the spurrite formation by solid-phase synthesis from SiO_2_ and calcite/CO_2_ or Ca_2_SiO_4_/Ca_3_SiO_5_, and calcite/CO_2_ at 880–910 °C [73,74].

During the decarbonation of samples in the evolving gases both Sulphur oxide (as SO_2_) and Carbon oxide (as CO_2_) are registered (Figure 9 and Figure 10). That suggests a solid-phase synthesis of sulphate-containing phases such as ternesite and/or anhydrite [75]. The confirmation of such assumptions was estimated by the solid residual PXRD analysis where ternesite was identified (Table 4). Ternesite is possible to form under high-temperature conditions in the presence of SiO_2_, CaO (solid phase), and sulphate ions (vapor). The sulphate vapor exerts partial pressure and partially binds to the oxides of the solid phase [24]. Like spurrite, ternesite can also formate by a solid-phase reaction of oxides (CaO, SiO_2_) and/or silicates (CaSiO_3_, Ca_2_SiO_4_), and anhydrite. The ternesite and/or anhydrite have existed in the solid residual (Table 4). That is why the final temperature of the experiment is 1100 °C, i.e., the temperature of their thermal decomposition was not reached [76].

### 4.4. Reaction Mechanism of Thermal Decomposition of Decorative Cement Composites

Based on the experimental results, their analyzes, and comparison with literature data, a generalized scheme of the chemistry of thermal reactions in the studied samples can be presented in terms of their thermal decomposition up to 1100 °C as follows:

I^st^ Range—Dehydratation (40–250 °C)

Ca_6_Al_2_(SO_4_)_3_(OH)_12_.26H_2_O → Ca_4_A1_2_(OH)_12_SO_4_.8H_2_O + 2CaSO_4_.0.5H_2_O + 17H_2_O (12)

2CaSO_4_.2H_2_O → 2CaSO_4_.0.5H_2_O + 3H_2_O (13)

Ca_4_Al_2_(OH)_12_(CO_3_).5H_2_O → CaAl_2_O_3_(CO_3_).3H_2_O + 3Ca(OH)_2_ + 5H_2_O (14)

2CaSO_4_.0.5H_2_O → 2CaSO_4_ + H_2_O (15)

CaAl_2_Si_3_O_10_.3H_2_O → CaAl_2_Si_3_O_10_.2H_2_O + H_2_O (16)

II^nd^ range—Dehydroxylation (420–470 °C)

Ca(OH)_2_ → CaO + H_2_O (17)

Ca_2_SiO_3_(OH)_2_ → CaSiO_3_ + CaO + H_2_O (18)

Ca_6_Si_6_O_17_(OH)_2_ → 6CaO + 6SiO_2_ + H_2_O (19)

III^rd^ range—Incomplete Dehydroxylation, Decarbonation and Desulphuration (520–730 °C)

CaAl_2_Si_3_O_10_.0.5H_2_O → CaAl_2_Si_3_O_10_ + 0.5H_2_O (20)

CaAl_2_O_3_(CO_3_).3H_2_O → CaCO_3_ + Al_2_O_3_ + 3H_2_O (21)

Ca_4_Al_2_(OH)_12_(SO_4_).8H_2_O → CaSO_4_ + 3CaO + Al_2_O_3_ + 14H_2_O (22)

Ca_4_Al_2_(OH)_12_(CO_3_).5H_2_O → CaCO_3_ + 3CaO+ Al_2_O_3_ + 11H_2_O (23)

CaAl_2_Si_3_O_10_.0.5H_2_O → CaSiO_3_ + 2SiO_2_ + Al_2_O_3_ + 0.5H_2_O (24)

Ca(HCO_3_)_2_ → CaCO_3_ + CO_2_ + H_2_O (25)

Ca(HSO_4_)_2_ → CaSO_4_ + SO_2_ + H_2_O + ½ O_2_ (26)

IV^th^ range—Incomplete Decarbonation and Desulphuration (730–850 °C)

CaMg(CO_3_)_2_ → CaO + MgO + 2CO_2_ (27)

CaCO_3_ → CaO + CO_2_ (28)

2CaSiO_3_ + 3CaO +CO_2_ → Ca_5_(SiO_4_)_2_(CO_3_) (29)

2CaSiO_3_ + 3CaO +SO_3_ → Ca_5_(SiO_4_)_2_(SO_4_) (30)

or/and

CaCO_3_ + 2SiO_2_ + 4CaO → Ca_5_(SiO_4_)_2_(CO_3_) (31)

CaCO_3_ + 2SiO_2_ + 4CaO → Ca_5_(SiO_4_)_2_(CO_3_) (32)

Solid-phase synthesis:

CaSiO_3_ + CaO → Ca_2_SiO_4_ (33)

2CaSiO_3_ + MgO → Ca_2_MgSiO_7_ (34)

2CaO + Al_2_O_3_ → Ca_2_Al_2_O_5_ (35)

CaSiO_3_ + Al_2_O_3_ + SiO_2_ → CaAl_2_Si_2_O_8_ (36)

## 5. Conclusions

The investigations provide new results on crystal-chemical and thermal properties of cement composites with high content of marble powder aggregate and reduced water-cement ratio. Powder X-ray diffraction and Fourier transform infrared spectroscopy prove the redistribution of anionic groups CO_3_^2−^, SO_4_^2−^, SiO_4_^4−^, AlO_4_^5−^, and OH^−^ (as O-H bond in structural OH- anions and O-H bond belonging to crystal bonded water molecules) from raw minerals to newly formed. The formation of cabroaluminates during hydration under the influence of marble powder has been established. Based on this investigation, the scheme of sample hydration has been defined. 

The thermal analysis with analysis of evolving gases defined four temperature ranges of sample decomposition: I^st^ range—dehydration (40–250 °C), II^nd^ range—dehydroxylation (420–470 °C), III^rd^ range—dehydroxylation, decarbonation, and desulphuration (520–730 °C), and IV^th^ range—decarbonation and desulphuration (730–850 °C). The lower temperature of decarbonation and desulphuration at the IIIrd range establishes the defects in solids, defined by CO_3_^2−^, and OH^−^, and SO_4_^2−^ incorporation. They are well presented in sample B and low pronounced in sample C with added HRWR water reducer. Such defects leading to structural compaction and physical-mechanical properties improvement. The formation of new solids (spurite and ternesite) under the influence of the partial pressure of evolving gases CO_2_ and SO_2_ at high temperatures in the process of thermal decomposition has been proven. The presence of calcium and silicon oxides in the solid phase has also been proven.

The obtained new results allowed: (i) the creation of complex chemical reactions, which describe the processes of hydration and thermal decomposition of the studied cement composites, and (ii) establishment differences in the reaction mechanisms depending on the use of cement replacement material (sand and marble powder). Finally, based on obtained results, the generalized scheme of sample thermal decomposition has been defined. 

The results obtained can be used to study the structure and properties of building materials and artifacts: ancient mortars related to the discovery of ancient composite production technologies and environmental knowledge in various archeological epochs.

## Figures and Tables

**Table 1:** Chemical and mineral composition of white Portland cement and used aggregates.

No	Chemical Composition of White Portland Cement—CEM I 52.5 N (wt%)										
1	SiO_2_		Al_2_O_3_	Fe_2_O_3_	CaO	MgO	Na_2_O	K_2_O		Free Lime	
	24.3		2.1	0.2	68.3	0.3	0.13	0.02		1.9	
	The mineral composition of white Portland cement, (wt%)										
2	Belite (C_2_S)			Alite (C_2_S)		Tricalcium aluminate (C_3_A)		Calcium aluminoferrite (C_4_AF)			
	72.13			15.28		5.23		0.61			
	Chemical composition of marble powder (wt%)										
3	CO_2_ + H_2_O	SiO_2_		Al_2_O_3_	Fe_2_O_3_	CaO	MgO	Na_2_O	K_2_O		MnO
	45.7	0.12		0.38	0.14	32.9	20.0	0.05	0.19		0.01
4	Chemical composition of sand (wt%)										
	Sand (SiO_2_)						over 85.0%				

**Table 2:** Compositions of the samples.

Sample	Aggregate	Ratio		
		Cement-to-Aggregate	Water-to-Cement	Water-to-Fines *
A028, A120	Sand	1:3	0.50	0.500
B028, B120	Marble powder	1:2	0.60	0.353
C028, C120	Marble powder	1:2	0.40 + HRWR	0.235

* all particles with sizes below 125 μm.

**Table 3:** Results from PXRD analysis of raw materials (white Portland cement, marble powder, and sand) and A028, A120, B028, B120, C028, and C120.

No	Description	Sample	Identified Phases (Name, ICDD Card Number; Formula)
1.	Minerals of raw materials and their relicts in samples		
	white Portland cement, A028, A120; B028, B120; C028, C120		Belite (C_2_S), #49-1673—Ca_2_SiO_4_
			Alite (C_3_S), #11-0593—Ca_3_SiO_5_
			Calcite, #47-1743—CaCO_3_
	white Portland cement		Tricalcium aluminate (C_3_A), #38-1429—Ca_3_Al_2_O_6_ Gypsum, #33-0311—CaSO_4_x2H_2_O
	marble powder, B028, B120; C028, C120		Dolomite, #36-0426—CaMg(CO_3_)_2_
			Mg-rich Calcite, #43-0697—CaCO_3_
	A028, A120		Quartz, #46-1045—SiO_2_
2.	Newly formed phases		
2.1.	hydrate phase	A028, A120 B028, B120 C028, C120	Portlandite (CH), #44-1481—Ca(OH)_2_
2.2.	C-S-H gel phases	A028, A120 B028, B120 C028, C120	Hillebrandite, #29-0373, #42-0538—Ca_2_SiO_3_(OH)_2_
		A028, A120	Scolecite, #41-1355—CaAl_2_Si_3_O_10_.3H_2_O
		A120 C028	Xonotlite, #23-0125—Ca_6_Si_6_O_17_(OH)_2_
2.3.	OH^−^ and HCO_3_^−^/CO_3_^2−^ phases	B028, B120 C120	Monocarboaluminate, #41-0219— Ca_4_Al_2_(OH)_12_(CO_3_)x5H_2_O
		B028 C028	Hemicarboaluminate, #41-0221—Ca_4_Al_2_(OH)_12_(OH)(CO_3_)_0.5_.4H_2_O
		B028, B120 C028, C120	Artinite, #72-1320—Mg_2_(CO_3_)(OH)_2_.3H_2_O
2.4.	OH^−^ and HSO_4_^−^/SO_4_^2−^ phases	A028, A120 B028, B120 C028, C120	Ettringite, #41-1451—Ca6Al_2_(SO_4_)3(OH)12·26H_2_O
		B028, B120 C028, C120	Calcium hydrogensulphate, #85-1271—Ca(HSO_4_)_2_
		A028, A120	Monosulphoaluminate, #50-1607—Ca_4_Al_2_(OH)_12_(SO_4_)x6H_2_O

**Table 4:** Results from PXRD analysis of A028, A120, B028, B120, C028, and C120 after heating up to 1100 °C.

No	Sample	Identified Phases (Name, ICDD Card Number; Formula)
1.	A028, A120; B028, B120 C028, C120	Larnite, #49-1673—Ca_2_SiO_4_
		Wollastonite, #42-0550—CaSiO_3_
		Anorthite, #41-1486—CaAl_2_Si_2_O_8_
	A028, A120 B120 C028, C120	Anhydrite, #37-1496—CaSO_4_
	A028, A120 B028, B120 C028	Ternesite #49-1807—Ca_5_(SiO_4_)_2_SO_4_
	A028, A120;	Quartz, #46-1045—SiO_2_
2.	B028, B120 C028, C120	Periclase, #45-0946—MgO
		Lime, #37-1497—CaO
		Akermanite, #35-0592—Ca_2_MgSi_2_O_7_
		Calcium Aluminium Oxide, #33-0252—Ca_2_Al_2_O_5_
		Spurrite, #13-0496—Ca_5_(SiO)_2_(CO_3_)

**Table 5:** Results from FTIR spectroscopy of raw materials (white Portland cement and marble powder) and A028, A120, B028, B120, C028, and C120.

Description/ References	Bond	ν_1_ (cm^−1^)	ν_2_ (cm^−1^)	ν_3_ (cm^−1^)	ν_4_ (cm^−1^)	Raw Material/ Sample
**1. Minerals of raw materials and their relicts in the samples**						
Belite (C_2_S), Ca_2_SiO_4_ Alite (C_3_S), Ca_3_SiO_5_ [17,27,28,29]	Si-O in SiO_4_^4−^	520		923	657	white Portland cement *, A028, A120, B028, B120, C028, C120
Tricalcium aluminate (C_3_A), Ca_3_Al_2_O_6_ [27,30]	Al-O in AlO_4_^5-^	754	435	856	545	white Portland cement
Calcite, CaCO_3_ [27,28,29,31]	C-O in CO_3_^2−^		875	1411 1473	713	white Portland cement *, A028, A120, B028, B120, C028, C120
Gypsum, CaSO_4_.2H_2_O [28,32,33]	S-O in SO_4_^2−^	995	457	1103, 1126, 1141	597, 669	white Portland cement
	O-H structural ^(1)^	3403		3552		
	O-H crystal ^(2)^		1617	3729		
Dolomite, CaMg(CO_3_)_2_ [27,28,29,31]	C-O in CO_3_^2−^		879	1442	727	marble powder *, B028, B120, C028, C120
Mg-rich Calcite, CaCO_3_ [28,29]	C-O in CO_3_^2−^	1081	879	1488	727	marble powder *, B028, B120, C028, C120
Quartz, SiO_2_ [28,29]	Si-O in SiO_2_	692	458	1083; 1172	512	sand, A028 *, A120
**2. Newly formed phases**						
**2.1. Hydrated phase**						
Portlandite (CH), Ca(OH)_2_ [28,29]	Ca-O-H	3642	1623	3743; 3245		A028 *, A120, B028, B120, C028, C120
	O-H structural^(1)^	3426		3556		
**2.2. C-S-H gel phases—hydrosilicates formed from main oxides: CaO, Al_2_O_3_, SiO_2_**						
Hillebrandite, Ca_2_SiO_3_(OH)_2_ [28,29]	Si-O in SiO_3_^2−^	964		1029; 1052		A028, A120 B028*, B120, C028, C120
	Ca-O-H	3642	1656	3245		
	O-H structural ^(1)^	3424		3540		
Scolecite CaAl_2_Si_3_O_10_.3H_2_O [28,29]	Al-O in AlO_6_^9^^-^	713		856	514 582 644	A028 *, A120
	Si-O in SiO_4_^4−^		458	1052; 1083; 1207; 1216		
	O-H crystal ^(2)^	3423	1623	3743; 3245		
Xonotlite—Ca_6_Si_6_O_17_(OH)_2_ [28,29]	Ca-O-H	3640	1621	3733; 3247		C028 *
	O-H structural ^(1)^	3424		3540		
	Si-O in SiO_4_^4−^		458	1041; 1052; 1083		
**2.3. OH^−^ and HCO_3_^−^/CO_3_^2−^ phases**						
Monocarboaluminate Ca_4_Al_2_(OH)_12_(CO_3_).5H_2_O [28,29]	Ca-O-H	3642	1691	3731; 3247		B120, C120 *
	O-H structural ^(1)^	3442		3561		
	Al-O in AlO_6_^9-^			856	511 667	
	C-O in CO_3_^2−^	1081	877	1444; 1481	700	
Hemicarboaluminate Ca_4_Al_2_(OH)_13_(CO_3_)_0.5_·5.5H_2_O [28,29]	Ca-O-H	3642	1691	3731; 3247		B028, C028 *
	O-H structural ^(1)^	3442		3548		
	Al-O in AlO_6_^9-^			856	511 661	
	C-O in CO_3_^2−^	1083	879	1452; 1481	700	
Artinite, Mg_2_(CO_3_)(OH)_2_.3H_2_O [34]	C-O in CO_3_^2−^	1083	877	1413	711 728	B028, B120, C028 *, C120
	O-H crystal ^(2)^		1627	3731; 3235		
	O-H structural ^(1)^	3430		3548		
**2.4. OH^−^ and HSO_4_^−^/SO_4_^2−^ phases**						
Ettringite, Ca6Al_2_(SO_4_)_3_(OH)12·26H_2_O [27,28,29,35,36,37]	Ca-O-H	3642	1643	3731; 3255		A028, A120, B028, B120 *, C028, C120
	O-H structural ^(1)^	3428		3561		
	S-O in SO_4_^2−^	1002		1139	599 667	
	Al-O-H		416	856		
Monosulphoaluminate, Ca_4_Al_2_(OH)_12_(SO_4_).6H_2_O [27,28,29,37]	Ca-O-H	3640	1621	3743; 3245		A028 *, A120
	O-H structural ^(1)^	3423		3556		
	S-O in SO_4_^2−^	1002	458	1099; 1147	605	
	Al-O in AlO_6_^9-^			856, 1031	514 534 671	
Calcium hydrogensulphate Ca(HSO_4_)_2_ [28,29,38]	O-H structural ^(1)^	3430		3548		B028, B120, C028*, C120
	S-O in SO_4_^2−^	1004	468	1105; 1130	599	

* The presented ν (cm^−1^) values are referred to the underlined and star-marked sample. Please note that the specified vibrations types are valid and the other listed samples and their exact ν (cm^−1^) values are shown in the Figure 5 and Figure 6. O-H structural ^(1)^—O-H bond in structural OH^−^ anion [32,33]. O-H crystal ^(2)^—O-H bond belonging to crystal bonded water molecules [32,33].

**Table 6:** Thermal decomposition (T_infl._) and ML of the raw materials and samples.

Sample	Ist Range		IInd Range		IIIrd Range		IVth Range		Total Mass Loses (%)
	40–200 (°C)		420–470 (°C)		520–730 (°C)		730–850 (°C)		
	Dehydratation of C-S-H Phases		Dehydroxylation of Ca(OH)_2_		Dehydroxylation of C-S-H, Uncomplete Decarbonation and Desulphurization of Ca(HCO_3_)_2_, Ca(HSO_4_)_2_		Uncomplete Decarbonation of MgCa(CO_3_)_2_, CaCO_3_		
	T_infl._ (°C)	Mass Losses (%)	T_infl._ (°C)	Mass Losses (%)	T_infl._ (°C)	Mass Losses (%)	T_infl._ (°C)	Mass Losses (%)	
white Portland cement	65.1 98.0	0.30 0.39	429.9	0.25	539.6	0.15	711.8	3.68	5.19
Marble powder	-	-	-	-	575.7	1.30	745.0 788.5 822.4 835.9	8.40 14.69 15.85 7.22	47.83
A028	108.5 - 177.1	1.01 - 0.52	465.3	0.68	596.7 625.1 682.8	1.46 1.90 1.00	742.5	3.33	11.44
A120	100.4 130.2 169.5	0.68 0.36 0.44	479.2	0.56	562.9 - 675.7	2.46 - 1.82	743.3 764.6	1.87 0.59	10.48
B028	85.2 116.2 -	0.50 0.82 -	469.0	0.91	529.5 - 685.5	4.15 - 1.68	734.7 794.3 814.4 827.3	4.49 11.91 8.69 5.95	42.80
B120	89.1 - -	1.10 - -	472.0	0.62	560.0 - 703.2	4.23 - 3.00	754.0 775.1 806.9 827.2	3.24 9.87 12.49 4.92	41.85
C028	94.2 - -	0.98 - -	472.6	0.46	585.5 - 693.5	1.34 - 1.31	798.4 816.7 827.9 830.5	6.21 10.03 8.80 2.86	38.81
C120	89.1 - -	1.09 - -	469.3	0.69	551.2 - 679.8	4.13 - 2.21	735.6 779.7 802.0 817.4	6.50 9.30 10.04 4.22	40.70

T_infl_._—_Temperature of the point of inflection.

## Abbreviations

Abbreviation	Description
A	white Portland cement + sand + water
B	white Portland cement + marble powder + water
C	white Portland cement + marble powder + HRWR + water
A028	Sample A after water curring of 28 days
A120	Sample A after water curring of 120 days
B028	Sample B after water curring of 28 days
B120	Sample B after water curring of 120 days
C028	Sample C after water curring of 28 days
C120	Sample C after water curring of 120 days
C-S-H	calcium silicate hydrate
HRWR	polycarboxylate-based admixture
PXRD	Powder X-ray diffraction
FTIR	Fourier Transformed Infrared
TG/DTG–DSC	Thermal analysis
ML, ΔG_total_	Mass Losses, ΔG
T_infl_	Temperature of the point of inflection
RT	Room temperature
EnT	Endothermal effect
ExT	Exothemal effect
